# *Bacillus* as a potential diagnostic marker for yellow tongue coating

**DOI:** 10.1038/srep32496

**Published:** 2016-08-31

**Authors:** Juan Ye, Xueting Cai, Jie Yang, Xiaoyan Sun, Chunping Hu, Junquan Xia, Jianping Shen, Kelei Su, Huaijiang Yan, Yuehua Xu, Yiyan Zhang, Sujie Zhang, Lijun Yang, Hao Zhi, Sizhi Paul Gao, Qiang Yu, Jingqing Hu, Peng Cao

**Affiliations:** 1 Affiliated Hospital of Integrated Traditional Chinese and Western Medicine, Nanjing University of Chinese Medicine, Nanjing 210028, China; 2 Laboratory of Cellular and Molecular Biology, Jiangsu Province Academy of Traditional Chinese Medicine, Nanjing 210028, China; 3 Department of Medicine, Memorial Sloan-Kettering Cancer Center (MSKCC), New York 10065, USA; 4 Department of Pharmacology, Shanghai Institute of Materia Medica, Chinese Academy of Sciences, Shanghai 201203, China; 5 Institute of Basic Theory of Traditional Chinese Medicine, China Academy of Chinese Medicine Sciences, Beijing 100700, China

## Abstract

Observation of tongue coating, a foundation for clinical diagnosis and treatment in traditional Chinese medicine (TCM), is a major indicator of the occurrence, development, and prognosis of disease. The biological basis of tongue diagnosis and relationship between the types and microorganisms of tongue coating remain elusive. Thirteen chronic erosive gastritis (CEG) patients with typical yellow tongue coating (YTC) and ten healthy volunteers with thin white tongue coating (WTC) were included in this study. Patients were provided a 2-course targeted treatment of a herbal medicine Ban Xia Xie Xin decoction, traditionally prescribed for CEG patients with YTC, to evaluate the relationship between tongue coating microbiota and diagnosis of CEG with typical YTC. The tongue coating segregation structure was determined using Illumina Miseq sequencing of the V4–V5 region of the 16S ribosomal RNA gene. *Bacillus* was significantly observed only in CEG patients with YTC, but not in patients who received the decoction. YTC (n = 22) and WTC (n = 29) samples were collected for bacterial culturing to illustrate the relationship between *Bacillus* and YTC. The *Bacillus* positivity rate of YTC samples was 72.7%; *Bacillus* was not observed in WTC samples. In conclusion, *Bacillus* was strongly associated with YTC.

Traditional Chinese medicine (TCM) is different with modern medicine include diagnostic methods and diagnostic index based on different cultural origins (Fig. 1A). Under the guidance of ancient philosophy, the theoretical system of TCM is gradually formed by integrating natural science and long-term medical practice. The tongue diagnosis, one of the distinctive diagnostic methods, has a very long history and plays an important role in TCM. The meaning of the tongue diagnosis can be found in the “Zhen Ji Tongue” records as early as during the Yin dynasty of China (about 4000 years ago). During the Eastern Han dynasty (about 2000 years ago), further development of the tongue diagnosis and the introduction of the word “tongue” were recorded in the medical sage Zhang Zhong jing’s “Treatise on”.

For thousands of years, practitioners have diagnosed the health status of a patient by inspecting the tongue, in particular, tongue surface patterns (Fig. 1B). Observing changes in tongue coating to infer systemic disorders involving internal organs as a method of clinical diagnosis is a unique feature of TCM. According to TCM theory: “A disease must come with a tongue coating manifestation. The coating is the outer manifestation, firmly corresponding to the inner disease, is the first clue for doctors to make a diagnosis: this cannot be neglected”^1^.Compared with other diagnostic regimens used in conventional medicine, tongue diagnosis may be more amenable and convenient for patients and doctors. Therefore, exploring the molecular basis of tongue diagnosis is critical for understanding this long-standing medical practice, and may provide a promising contribution to personalized medicine^2^.

In recent studies, it has been verified that the microbiome of the human body is associated with human physiology and pathology^3^. Researchers have shown that the oral microbiome is associated with many diseases, such as pancreatic diseases^4^,^5^, pediatric inflammatory bowel disease^6^, coronary heart disease^7^, rheumatoid arthritis^8^, and gastrointestinal cancer^9^. It has also been reported that the relationship between the microbiome of tongue coating and TCM diagnosis was relevant to TCM syndrome differentiation. For example, cold syndromes are pathological changes and symptoms caused by exogenous pathogenic cold or constitutional yang deficiency and hot syndromes are caused by exogenous pathogenic heat or constitutional yin deficiency (Generally, symptoms characterized by excitation, restlessness, hyperactivity, and optimism belong to yang syndromes, while those characterized by inhibition, quietude, decline, and gloominess are yin.). Cold and hot syndromes are two contrary, but inner-related, conditions that appear in the human body and have been used to characterize a patient’s status in many diseases or conditions that may exhibit different microbiota patterns in the tongue coating^10^. However, the relationship between particular tongue coating types and changes in microorganisms during treatment is currently unknown.

Based on TCM theory, the tongue is an outer extension of *Pi* (spleen-pancreas) and *Wei* (stomach). However, the tongue coating is produced by the *Wei Qi* (Defense *Qi*) through fumigation^11^. Any herbal prescription for treatment must correspond to the specific disease and syndrome category. The tongue coating is classified into three categories based on different color types, namely, white tongue coating (WTC), yellow tongue coating (YTC), and black and grey tongue coating. A YTC is deemed as a sign of internal retention of dampness and heat. YTC must be considered in the differential diagnoses of patients suffering from certain types of diseases. Chronic erosive gastritis (CEG) is a very common gastrointestinal disease in China^12^. Patients with CEG always present with various gastrointestinal tract symptoms and histological changes in gastric mucosa leading to decreased quality of life^13^. Currently, TCM practitioners often use Chinese herbal medicines to successfully treat gastrointestinal disorders categorized as damp-heat syndrome (i.e., CEG, enteritis, gastric ulcer, and gastralgia), which is mainly characterized by YTC^14^. The Ban Xia Xie Xin decoction was created by the ancient TCM guru named Zhang Zhong Jing to treat abdominal stuffiness syndrome (*Pi Zheng*). The formula was first described about 1800 years ago in the Chinese herbal medicine classic book of Shang Han Lun (“On Cold Damage”)and used extensively for gastrointestinal disease. The common clinical use of Ban Xia Xie Xin decoction is according to the main clinical features of hot and humid, moss yellow, mouth bitter, and noisy^15^.

In this study, we conducted a next-generation sequencing analysis of YTC microbiome samples from 13 CEG patients receiving the Ban Xia Xie Xin decoction during 2-treatment periods and 10 thin-WTC samples from healthy volunteers to find the precise microbiome of YTC. Furthermore, we collected tongue coating samples from patients with other diseases and control healthy volunteers, divided the samples by tongue coating colors (i.e., yellow and white) and conducted microbial cultivation to validate the relationship between the precise microbiome and YTC. We hypothesized that a particular tongue coating microbiome may correlate with YTC, which will enable a diagnosis of YTC by detecting certain microbiota instead of subjective visual inspection. The findings of this study provide evidence, for the first time, for the biological basis of tongue diagnosis in TCM and contribute to the objective and scientific interpretation of TCM.

## Results

### Overall composition of tongue coating bacterial communities

**Figure 1:**
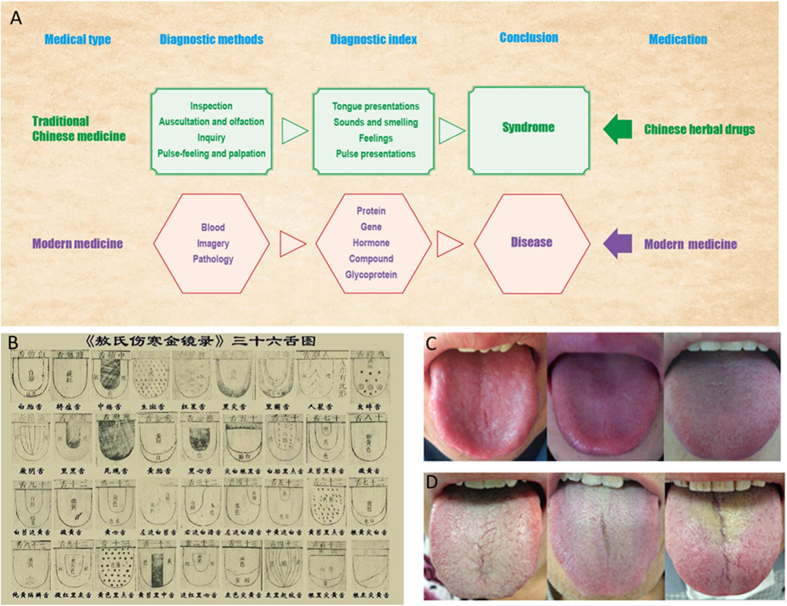
Differences in the diagnosis methods and treatment methods of traditional Chinese medicine and Western Medicine (**A**). An ancient instruction for tongue coating classification recorded in “Aoshi-Shanghan-jin-jing-lu”, a TCM book compiled in the Yuan Dynasty of China (1341 AD) (**B**), and the classification of typical tongue coat appearance in healthy individuals with a thin layer of white tongue coating (**C**) and chronic erosive gastritis patients with yellow tongue coating (**D**) are shown.

We analyzed 10 thin-WTC samples from 10 healthy subjects and 33 YTC samples from 13 CEG patients who received the Ban Xia Xie Xin decoction for 2 treatment courses (Fig. 1C,D; Supplementary Table S1). Single herb and Ban Xia Xie Xin decoction were determined by the extraction method and the HPLC method. *Guanosine*, *baicalin*, *6-gingerol*, *lobetyolin*, *monoammonium glycyrrhizinate*, *berberine*, *betulinicacidand oleanolicacid* were used as reference standard to detectthe constituents of *Pinellia ternata Breit*, *Scutellaria baicalensis Georgi*, *Rhizoma Zingiberis*, *Salvia miltiorrhiza Bunge*, *GLycyrrhiza uralensis Fisch*, *Coptis chinensis Franch*, *Ziziphus jujuba Mill* separately. Furthermore, we evaluated the ecological features of the tongue coating bacterial communities of the 2 groups by a variety of indices at the operational taxonomic unit (OTU) level, based on the 16S rRNA sequencing methods using the IlluminaMiseq platform^16^. In total, 974434 high-quality raw reads were obtained from all 43 samples. Sequences were assigned to species-level OTUs using a distance-based similarity of > 97%. A total of 230 OTUs were identified. The rarefaction curve plateaued with the sequencing shown in Supplementary Fig. S1A, and the Shannon diversity estimates of all samples already reached stable values at this sequencing depth, suggesting that most of the diversity had already been captured (Supplementary Fig. S1B).

**Figure 2:**
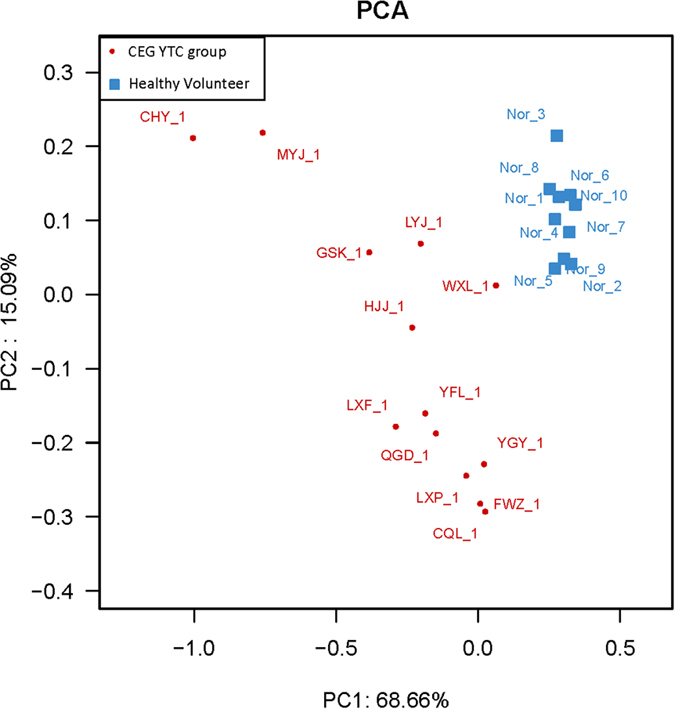
Principal component analysis plots of the tongue-coating microbiome diversity between samples from patients with chronic erosive gastritis yellow tongue coating and normal controls. The 230 operational taxonomic units presented in all samples were included in the analysis. Component 1 and 2 exhibited 68.66% and 15.09% of all variance, respectively.

In our initial analyses, we examined the global microbial diversity of the entire microbial population. There were no statistically significant differences in the diversity estimates obtained from species richness and evenness^17^ between CEG patients and healthy controls (Supplementary Fig. S2). In order to observe the CEG YTC sample clusters separately from the normal controls, we performed principal component analysis of the log-normalized abundance of the 230 OTUs. The distribution of samples in the space of the first and second main components of the principal component analysis accounted for 68.66% and 15.09% of the total variations, respectively (Fig. 2). The healthy controls and CEG patients were distributed in separate regions and there was a clear boundary between the patients and healthy controls, indicating that the 2 groups are distinguishable based on their OTUs.

### Characterization of YTC microbiota in CEG patients

**Figure 3:**
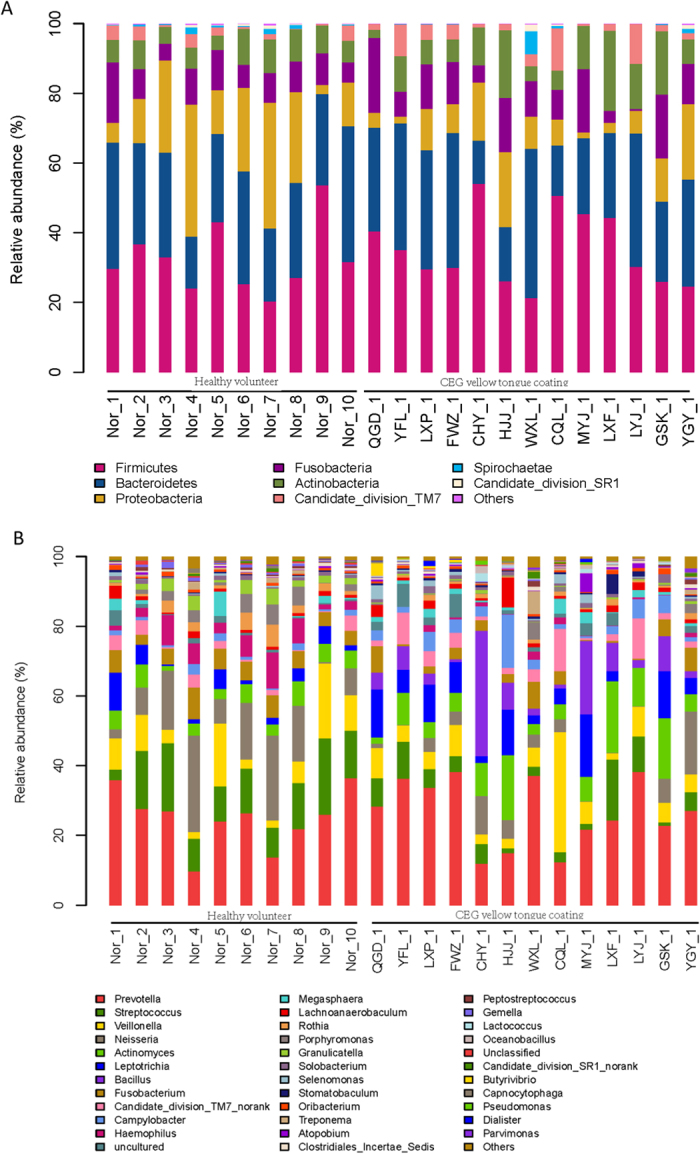
16S ribosomal ribonucleic acid sequencing analysis and taxonomy classification of the tongue-coating microbiome (normal control and chronic erosive gastritis yellow tongue coating group) at the phylum (**A**) and genus (**B**) levels.

By examining the results of the RDP classification algorithm^18^ at the phylum level, the tongue coating microbiota of the 2 groups were both dominated by 7 phyla (relative abundance ≥1%) according to our assessment. Among all bacterial groups revealed by interpretable sequences, Firmicutes was the most predominant phylum, contributing to 36 ± 1% of the tongue coating microbiota, followed by Bacteroidetes, (27.5 ± 0.5%), Proteobacteria (13.5 ± 4.5%), Fusobacteria (10 ± 1%), Actinobacteria (9 ± 2%), TM7 (3 ± 1%), and Spirochaeta (1 ± 0%) (Fig. 3A). Microbial compositions had small individual variability at the phylum level. The abundance of Proteobacteria in samples from the CEG YTC group was significantly lower compared to the healthy group. At the class level, there was a significant higher abundance of Epsilonproteobacteria and lower abundance of Betaproteobacteria and Gammaproteobacteria based on their OTUs (P < 0.05; Supplementary Fig. S3A).

**Figure 4:**
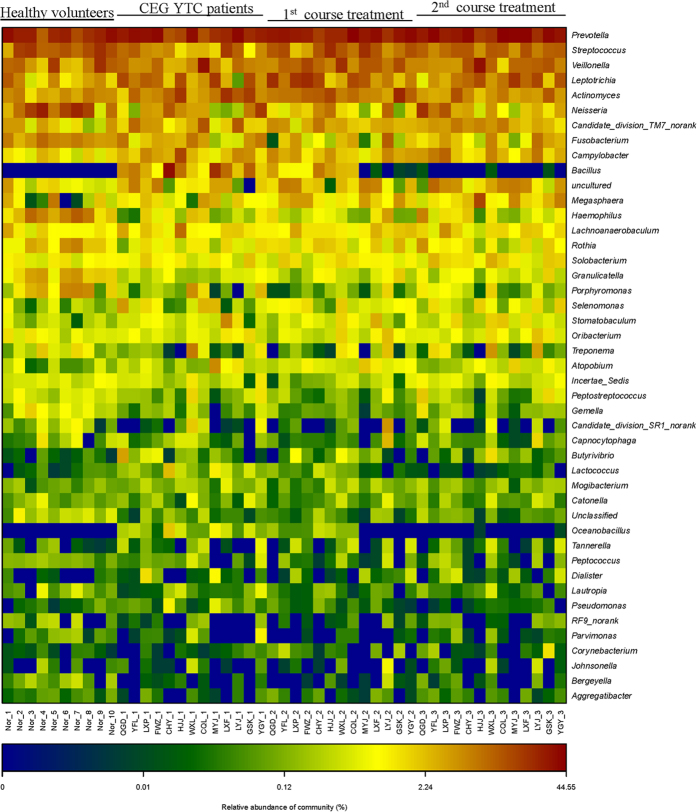
A heat map of the relative abundance of microbial taxa of the tongue coating microbiome characterized by 16S ribosomal ribonucleic acidgene sequencing of the 4 groups at the genus level.

An inter-individual variability was observed according to the 16S rRNA sequencing analysis and taxonomy-based comparisons of tongue coating microbiota at the genus level (Fig. 3B). However, remarkably, *Bacillus* was only found in the CEG YTC group in all 13 CEG patients compared to normal controls (Fig. 4). The abundance of *Campylobacter* was significantly higher, but the abundance of 8 genera (i.e. *Fusobacterium, Gemella, Granulicatella, Haemophilus, Neisseria, Porphyromonas, Rothia, and Streptococcus*) was significantly lower in the CEG YTC group compared to the normal controls (P < 0.05; Supplementary Fig. S3B).

**Figure 5:**
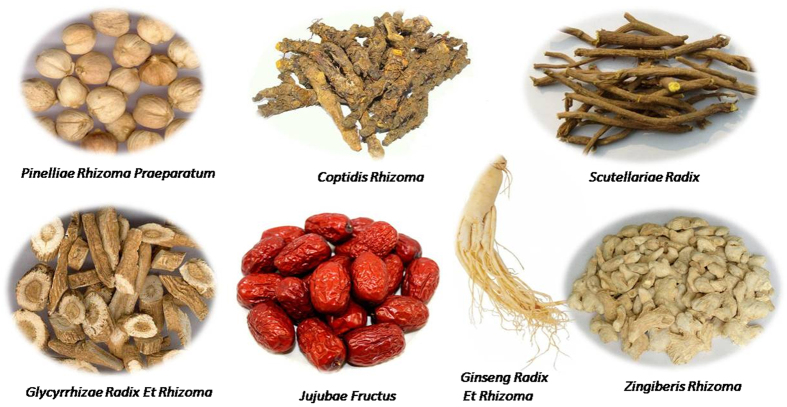
The seven herbs in Ban Xia Xie Xin decoction.

**Figure 6:**
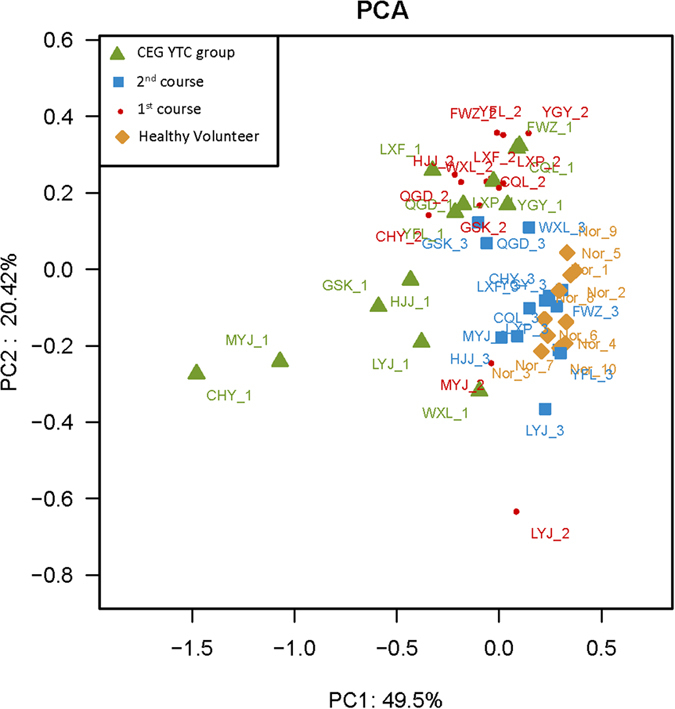
Principal component analysis plots of the tongue-coating microbiome diversity among samples from the 43 cases. Green triangles: chronic erosive gastritis (CEG) patients with yellow tongue coating; red circles: first course treatment of Ban Xia Xie Xin decoction for CEG patients with yellow tongue coating; blue squares: second course treatment of Ban Xia Xie Xin decoction for CEG patients with yellow tongue coating; brown diamond : healthy controls.

In order to evaluate whether *Bacillus* was correlated with CEG YTC, we performed a 2-course treatment in patients with the Ban Xia Xie Xin decoction, a classic formula that has been administered for hundreds of years to treat YTC CEG patients. The Ban Xia Xie Xin decoction is composed of *pinellia tuber (Rz. Pinelliae Preparatum*), *baikal skullcap* root (*Rx. Scutellariae*), dried *ginger* root (*Rz. Zingiberis*), *ginseng* root (*Rx. Ginseng*), *liquorice* root (*Rx. Glycyrrhiza*), *coptis rhizome (Rz. Coptidis*), and *Chinese date (Fr. Jujubae*) (Fig. 5). Traditionally, this decoction is used to purge stomach “fire” and harmonize the stomach and intestines^14^,^19^. We initially evaluated the water extracts to ensure its authenticity, quality, and time stability by HPLC (Supplementary Fig. S4). According to HPLC analysis, aqueous extracts of Ban Xia Xie Xin placed at 4 °C for 7 days had almost identical chemical component peaks as the first day extracts. We then administered Ban Xia Xie Xin decoction to the 13 CEG patients in a 2-course treatment (7 days/course) to observe any changes in tongue coating microbiota by 16S rRNA sequencing. Overall, the phylum composition of the first and second treatment course groups was similar in the CEG YTC and healthy control groups (Supplementary Fig. S5). After receiving the Ban Xia Xie Xin decoction for the first 7 days, *Bacillus* decreased or completely disappeared from the tongue coating (Fig. 4). After the second course of treatment (day 14), almost all *Bacillus* had disappeared (Fig. 4). We also performed principal component analysis of the 4 groups (i.e., first and second treatment course groups, CEG group, and healthy controls) based on OTUs (Fig. 6). We found that the second treatment course group was easily separable from the CEG and first course groups, and similar to the healthy control group.

### *Bacillus* is a component of most YTC, but not WTC

To verify whether *Bacillus* was associated with YTC or a certain disease, we recruited patients in the gastroenterology (n = 26) and cardiovascular (n = 14) departments and other healthy volunteers (n = 11) and divided the participants into 2 groups by tongue coating color (i.e., yellow and white). According to Bergey’s Manual of Determinative Bacteriology^20^, we chose phenylalanine agar to inoculate the tongue coating samples. Patient information and the staining microscopy results are shown in Supplementary Tables S2–S3. Based on the statistical analysis, *Bacillus* was cultivated in 12 out of 16 cases from the gastroenterology department and 4 out of 6 cases from the cardiovascular department. The positive rate of *Bacillus* in all collected YTC samples was 72.7%; the WTC samples showed no signs of *Bacillus* (0%; Table 1). Therefore, we concluded that *Bacillus* was more likely to survive in YTC.

## Discussion

Tongue diagnosis is a unique method in TCM. In the long history of traditional clinical practice in China and other Eastern countries, syndrome classification is often used as a guideline for disease classification in TCM practice, and tongue coating appearance is one of the major criteria for diagnosis^21^. It is known that the microbiota colonizing the surfaces of human oral cavity play an important role in human health and disease or is relevant to syndrome differentiation^9^,^22^,^23^. Previous work compared the tongue coating microbiome in patients with Hot and Cold Syndromes and constructed their tongue microbiota-imbalanced networks^10^. However, we preferred to study one disease with one typical tongue coating, namely CEG patients with YTC, and treat patients with Chinese medicine to observe changes in tongue coating microflora during the treatment. The findings in this report support our hypothesis that particular tongue coating microbiome may correlate with YTC.

The tongue is considered as an extension of the upper gastrointestinal tract in both conventional medicine and TCM^24^. CEG is a very common gastrointestinal disease in China. In the discovery phase, we selected CEG patients with YTC, according to TCM, as study participants. To test our hypothesis, we conducted a 16S rRNA sequencing on the V4–V5 region^25^ via Illumina Miseq platform of the tongue coating from 13 consecutive CEG patients treated with a 2-course treatment of the Ban Xia Xie Xin decoction, and 10 healthy volunteers with thin WTCs.

We revealed differences in the tongue coating microbiota structure patterns in CEG patients with YTC compared to healthy volunteers. A potential connection between the tongue coating microbiome and traditional tongue diagnosis has been previously demonstrated. The significance of syndrome differentiating tongue coating microbiota has also been highlighted by a recent publication^10^. The groups of bacterial phyla in the tongue coating microbiota identified in our study, namely, Firmicutes, Bacteroidetes, Proteobacteria, Fusobacteria, and Actinobacteria, were consistent withprevious reports^26^,^27^. In healthy tongue coatings, we were able to confirm that Firmicutes, Proteobacteria, and Bacteroidetes were the most abundant phyla, followed by Fusobacteria and Actinobacteria. Similar findings have been previously reported in other studies as well^28^,^29^, which confirm the reliability and consistency of our study.

Interestingly, we determined that *Bacillus* was only found in CEG patients with YTC. Since methods for validating a bacterial genus were limited^30^, we questioned whether administering a targeted therapy to patients would clarify the correlation between *Bacillus* and CEG with YTC, a subtype of CEG deemed by the principle of TCM diagnosis. After 2 courses of treatment, *Bacillus* growth was abrogated, suggesting that *Bacillus* correlated with CEG YTC. The fact that the therapy reversed the tongue coating pattern in CEG patients compared to that of healthy controls strongly suggested that the tongue coating microbiome reflected disease status.

Based on our analysis, we revealed that the abundance of *Campylobacter* was higher in the CEG YTC group compared to healthy controls. High levels of *Campylobacter* have also been observed in pancreatic cancer patients; therefore, it is deemed as a pancreatic cancer diagnosis biomarker^4^. Furthermore, we detected decreased levels of *Rothia, Granulicatella, Streptococcus, Fusobacterium, Gemella, Haemophilus, Neisseria, and Porphyromonas* in CEG patients with YTC. All of these 8 genera have been described in normal populations of healthy oral microbiota^4^,^5^,^6^,^7^,^8^,^9^,^10^,^11^,^12^,^13^,^14^,^15^,^16^,^17^,^18^,^19^,^20^,^21^,^22^,^23^,^24^,^25^,^26^,^27^,^28^,^29^,^30^,^31^. An increasing trend in these bacteria was observed after therapy, albeit not statistically significant (Fig. S6). We deduced that the Ban Xia Xie Xin decoction might have a significant effect on the patients’ overall status and environment. A longer course of such therapy may yield a statistically significant outcome (i.e., the tongue coating microbiota would completely return to normal).

After the treatment, the tongue coating remained yellow although there was an improvement in the symptoms of CEG patients. It is still unknown whether the *Bacillus* in the YTC of CEG patients was the cause or the consequence of this tongue coating color change. Therefore, we performed a validation test using microbial cultivation. We found that *Bacillus* was one of the components in most YTC, but not in WTC. However, *Bacillus* was not the determinant for tongue coating color because the tongue coating can be yellow without the presence of *Bacillus*. We purport that although *Bacillus* is unrelated to a certain disease, it may reflect a common physiological state of the human body or a specific pathological pattern according to TCM diagnosis. Our study may provide the first line of evidence for the potential use of the tongue coating microbiota as a novel holistic biomarker for characterizing patient subtypes.

Nevertheless, our findings were restricted mainly to the genus level. The limitation for discernment at the species-level could potentially be attributed to the database and the type of primer that was used in sequencing studies. We also acknowledge that the average ages of the CEG YTC group and healthy volunteers were considerably different, and more investigation into the impact of age on the tongue microbiome is required in future research. Moreover, our findings can be potentially used to inform future studies involving the development of animal models to elucidate the mechanisms behind the disappearance of *Bacillus* after treatment with the Ban Xia Xie Xin decoction.

In summary, our findings in this report provide important clues regarding the relationship between *Bacillus* and YTC. We were also able to determine that tongue coating microbiota may manifest the status of a disease, assist syndrome differentiation, define treatment methods, guide herbal prescription, and/or determine disease prognosis. The observations described here shed light in a new direction for understanding the biological basis of tongue diagnosis in TCM and clues for syndrome differentiation.

## Material and Methods

### Study participants

This study was approved by the Jiangsu Province Hospital on Integration of Chinese and Western Medicine Institutional Ethics Review Board (2014LW001). Consecutive 13 CEG YTC patients from the Jiangsu Province Hospital on Integration of Chinese and Western Medicine were recruited into the trial using a western disease classification and then stratified into the appropriate TCM patterns for treatment. All participants in this study provided their informed consent. Participants with a confirmed diagnosis of CEG by endoscopy combined with pathologic biopsy^32^, and YTC, according to the National TCM Diagnosis Principle^33^, by TCM doctors were included in this study. All 13 patients were tested for *Helicobacter pylori* infection using the fast urea test and histology. All healthy volunteers were tested using the 14C urea breath test, reported no stomach discomfort in the past 1 year, and exhibited a normal tongue coating as judged by TCM doctors. Exclusion criteria included: participants who had antibiotic treatment and glucocorticoids within the last 3 months, or showing evidence of systemic disease that may influence oral health or pregnancy. The methods were conducted in accordance with the Jiangsu Province Hospital on Integration of Chinese and Western Medicine Institutional Ethics Review Board guidelines.

### Tongue coating samples

Tongue coating samples were obtained and photographed in the morning by one examiner. All participants were required to gargle sterile water 3times (10 mLper time) to remove food residue prior to sampling. Tongue coating samples were collected from the middle surface of the tongue dorsum using a sterile spatula by swabbing 3 times from back to front (approx. 2-cm-long swabbing motions)^34^. The samples were stored in liquid nitrogen immediately.

### DNA extraction and PCR amplification

Microbial DNA was extracted from tongue coating samples using the E.Z.N.A.® Soil DNA Kit (Omega Bio-tek, Norcross, GA, U.S.) according to manufacturer’s protocols. The V4–V5 region of the bacteria 16S ribosomal RNA gene were amplified by PCR (95 °C for 2 min, followed by 25 cycles at 95 °C for 30 s, 55 °C for 30 s, and 72 °C for 30 s, and a final extension at 72 °C for 5 min) using primers 515F 5′-barcode-GTGCCAGCMGCCGCGG)-3′ and 907R 5,-CCGTCAATTCMTTTRAGTTT-3 where barcode is an 8-base sequence unique to each sample. PCR reactions were performed in triplicate 20 μL mixtures containing 4 μL of 5× FastPfu Buffer, 2 μL of 2.5 mMdNTPs, 0.8 μL of each primer (5 μM), 0.4 μL of FastPfu Polymerase (Transgene, Beijing, China), and 10 ng of template DNA.

### Illumina MiSeq sequencing

Amplicons were extracted from 2% agarose gels and purified using the AxyPrep DNA Gel Extraction Kit (Axygen Biosciences, Union City, CA, U.S.) according to the manufacturer’s instructions and quantified using QuantiFluor™-ST (Promega, Wisconsin, U.S.). Purified amplicons were pooled in equimolar and paired-end sequenced (2 × 250) on an Illumina MiSeq platform according to the standard protocols. The raw reads were deposited into the NCBI Sequence Read Archive database.

### Processing of sequencing data

Raw fastq files were demultiplexed and quality-filtered using QIIME (version 1.17) with the following criteria: (i) 250 bp reads were truncated at any site receiving an average quality score <20 over a 10 bp sliding window, discarding the truncated reads that were shorter than 50 bp; (ii) exact barcode matching, 2 nucleotide mismatch in primer matching, and reads containing ambiguous characters were removed; (iii) only sequences that overlap >10 bp were assembled according to their overlap sequence. Reads that could not be assembled were discarded.

OTUs were clustered with 97% similarity cutoff using UPARSE^35^(version 7.1 http://drive5.com/uparse/) and chimeric sequences were identified and removed using UCHIME^36^. The phylogenetic affiliation of each 16S rRNA gene sequence was analyzed by RDP Classifier^37^ (http://rdp.cme.msu.edu/) against the silva (SSU115)16S rRNA database using a confidence threshold of 70% 22,38,39.

### Ban Xia Xie Xin Decoction preparation and quality control

All the herbs were purchased from Jiangsu Province Hospital on Integration of Chinese and Western Medicine (Nanjing, China). Firstly, mixture of crude herbs, *Pinellia Tuber* 12 g, *Radix Scutellariae* 9 g, *Dried Ginger* 9 g, *Pilose Asiabell Root* 9 g, *Liquorice Root* 9 g, *Rhizoma Coptidis* 3 g, and *Jujubae Fructus* 4 g were immersed separately in a 10-fold mass of water for 30 min. Secondly, 1000 mL of water was added after impregnation, the extract was heated for 45 min, and then the liquid was leached. Thirdly, 500 mL of water was added into the residue, the extract was heated for 45 min, and then the liquid was leached. Fourthly, the 2 parts of leaching liquid were merged for liquid static sedimentation, filtration, and concentration and packed for use; the samples were stored at 4 °C before use.

The HPLC experiment was performed on a serial System (LC-20AT HPLC system, Shimadzu Inc., Japan) with a SPD-20A detector using validated methods for linearity, limits of detection, quantification, reproducibility, and recovery. With pure compounds (purity ≥98%) from Shanghai Tauto Biotech Co., Ltd (Shanghai, China), these assays were confirmed to be accurate, reproducible, and sensitive. In brief, samples were separated on an Inertsil ODS-SP C18 column (250 mm × 4.6 mm, 5 μm). The mobile phase consisted of acetonitrile (A) and 0.1% aqueous acetic acid (B). The mobile phase flow rate was 1.0 mL/min, and the column temperature was controlled at 30 °C. A Shimadzu diode array detector was set at 280 nm to detect the constituents of the Ban Xia Xie Xin decoction.

### Bacterial culture and Morphological identification

Phenylalanine agar (NaCl, 5 g; yeast extract, 3 g; DL-phenylalanine, 2 g; Na_2_HPO_4_, 1 g; agar, 12 g; distilled water, 1 L; pH 7.3) was made and distributed in 200 mL conical flasks, autoclaved at 121 °C for 20 min, and then poured into plates. Tongue coating samples were diluted 1000 times, 100 μLof which were inoculated into the phenylalanine agar bases. Bacterial species were initially and roughly identified according to their growth situations. Inoculated agar bases were cultured in a 37 °C incubator. The morphology of the colonies was observed 48 h later. A single colony was placed on a fresh surface for further identification using a sterile pipette tip. The gram-staining kit used in this study was produced by Solarbio Co., Ltd (Beijing, China).

### Statistical analysis

A Students’ t-test was performed to test the significance among healthy control, CEG YTC, first course treatment, and second course treatment groups and a p-value < 0.05 value was considered to be statistically significant.

## Additional Information

**How to cite this article**: Ye, J. *et al. Bacillus* as a potential diagnostic marker for yellow tongue coating. *Sci. Rep.* **6**, 32496; doi: 10.1038/srep32496 (2016).

## Supplementary Material

Supplementary Information

## Figures and Tables

**Table 1:** Sample collection and *Bacillus* positive cases.

	Healthy volunteer (n = 11)		Patients from Gastroenterology Department (n = 26)		Patients from Cardiovascular Department (n = 14)	
	YTC	WTC	YTC	WTC	YTC	WTC
Number of cases	0	11	16	10	6	8
*Bacillus*(+)	0	0	12	0	4	0

YTC, yellow tongue coating; WTC, white tongue coating.
