# Supplementary material for: Bacillus as a potential diagnostic marker for yellow tongue coating
Source: Sci Rep. 2016 Aug 31;6:32496. doi: 10.1038/srep32496 (PMC5006162; doi:10.1038/srep32496)
Supplement: Supplementary Information [file srep32496-s1.pdf]

Bacillus as a potential diagnostic marker for yellow tongue coating

Supporting Information Appendix

Juan Ye[a,b], XuetingCai[a,b], Jie Yang[a,b], XiaoyanSun[a,b], Chunping Hu[a,b], JunquanXia[a,b], JianpingShen[a,b], Kelei Su[a,b], Huaijiang Yan[a,b],YuehuaXu[b], Yiyan Zhang[a,b],Sujie Zhang[a,b], LijunYang[a,b],HaoZhi[a,b],Sizhi Paul Gao[c],QiangYu[d],Jingqing Hu[e*], PengCao[a,b,e*]

[a] Affiliated Hospital of Integrated Traditional Chinese and Western Medicine, Nanjing University of Chinese Medicine, Nanjing 210028, China
[b] Laboratory of Cellular and Molecular Biology, Jiangsu Province Academy of Traditional Chinese Medicine, Nanjing 210028, China
[c] Department of Medicine, Memorial Sloan-Kettering Cancer Center (MSKCC), New York 10065, USA.
[d] Department of Pharmacology, Shanghai Institute of Materia Medica, Chinese Academy of Sciences, Shanghai201203, China
[e] Institute of Basic Theory of Traditional Chinese Medicine, China Academy of Chinese Medicine Sciences, Beijing 100700, China

Correspondence

Peng Cao
Laboratory of Cellular and Molecular Biology,
Jiangsu Province Academy of Traditional Chinese Medicine
100 Shizi Street, Hongshang Road
Nanjing, Jiangsu, 210028
China
Tel./fax: +86 25 85608666.
Email:pcao79@yahoo.com

Jingqing Hu
Institute of Basic Theory of Traditional Chinese Medicine,
China Academy of Chinese Medicine Sciences,
16 Nanxiao Street, Dongzhimen Road
Beijing, 100700
China
Tel./fax: +86 10 64014411
Email: gcp306@126.com

Supporting Figures

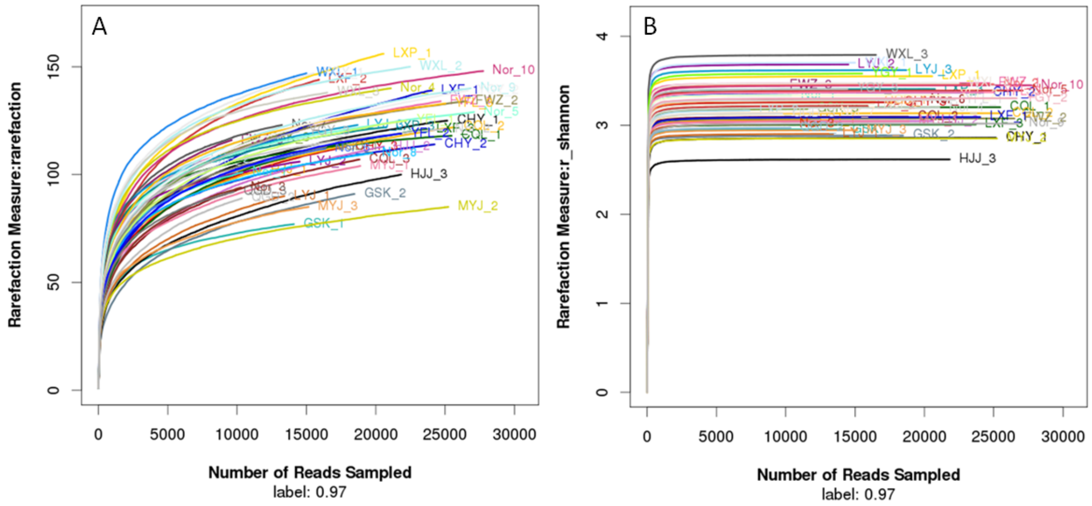

Fig.S1. Rarefaction curves (A) alpha diversity of all 43 samples based on species-level OTUs; (B) Shannon rarefaction curves of 43 samples.

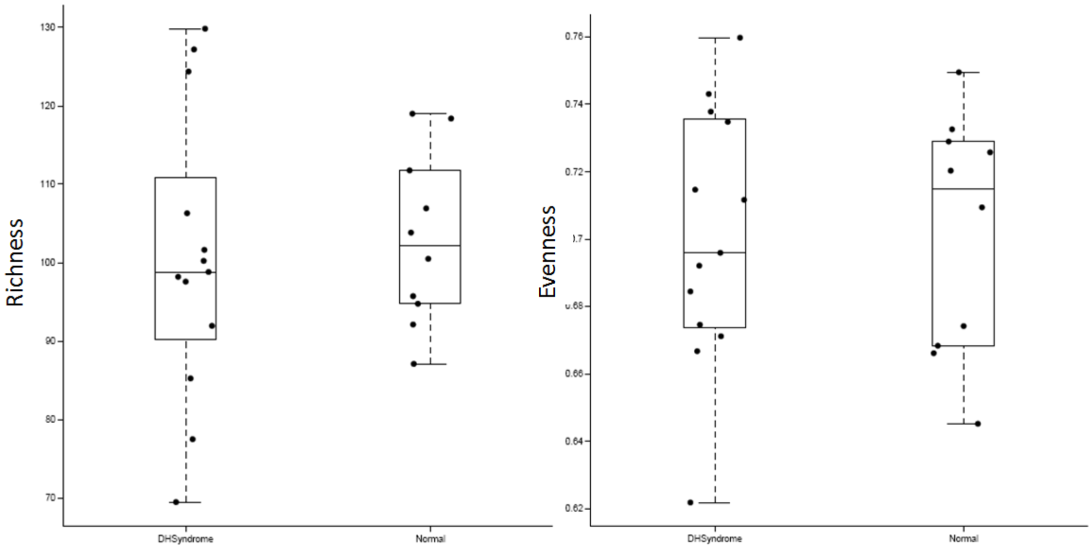

Fig.S2. Richness and evenness for the OUTs observed in this study for CEG yellow tongue caoting group (n=13) and healthy control (n=10) . By the Student's t-test, the two groups had no significant difference in richness and evenness(*P*=0.126 and *P*=0.381 respectively).

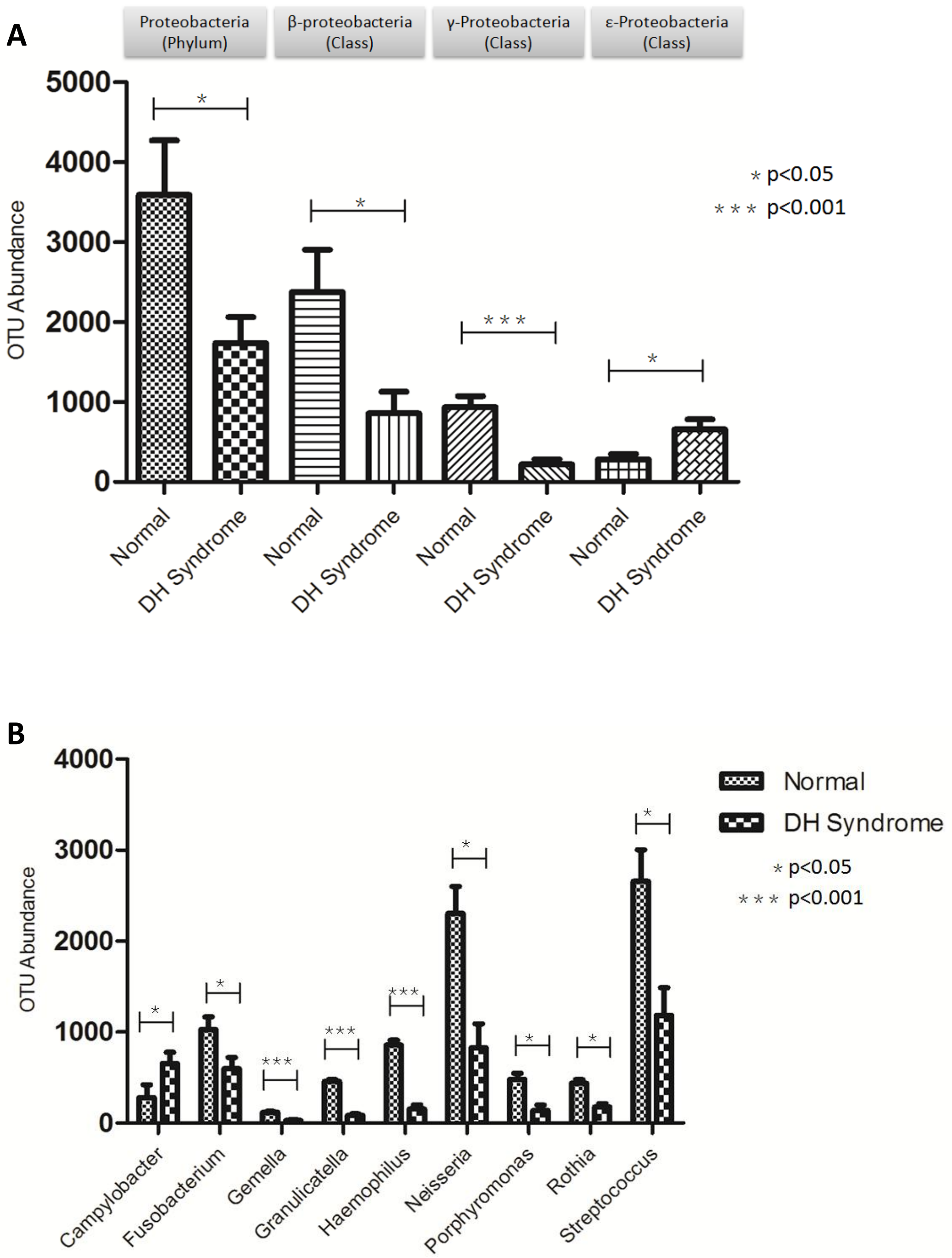

Fig.S3. Abundance of OTUs in patients with CEG yellow tongue coating and healthy controls. A:at Pylum level; B:at genus level Student's *t*-test was uesd; mean ± S.E.M. ("YTC":yellow tongue coating)

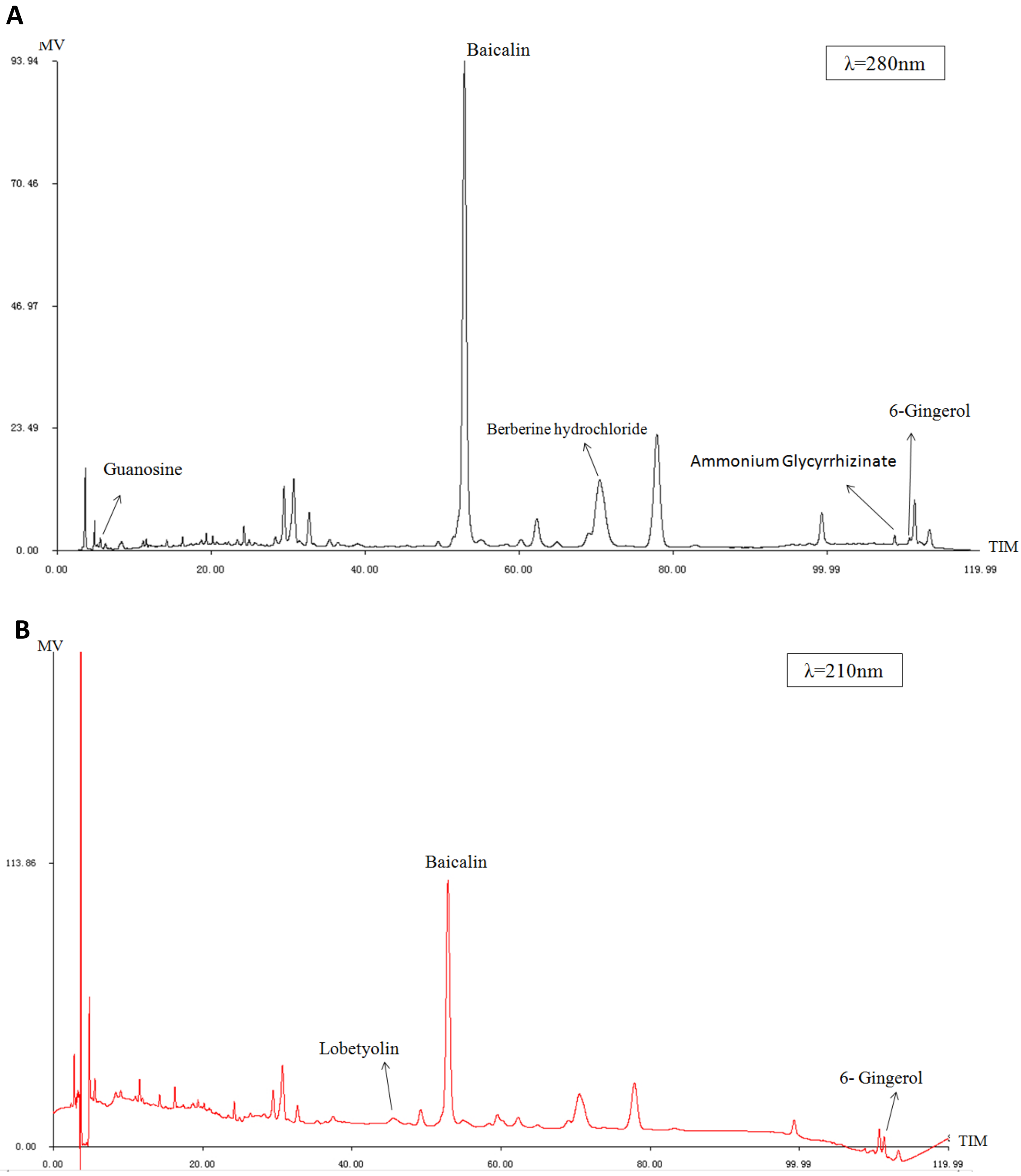
A
MV
93.94
70.46
46.97
23.49
0.00
Baicalin
λ=280nm
Berberine hydrochloride
Ammonium Glycyrrhizinate
6-Gingerol
Guanosine
TIM
0.00
20.00
40.00
60.00
80.00
99.99
119.99
B
MV
λ=210nm
113.86
Baicalin
Lobetyolin
6- Gingerol
0.00
TIM
0.00
20.00
40.00
60.00
80.00
99.99
119.99

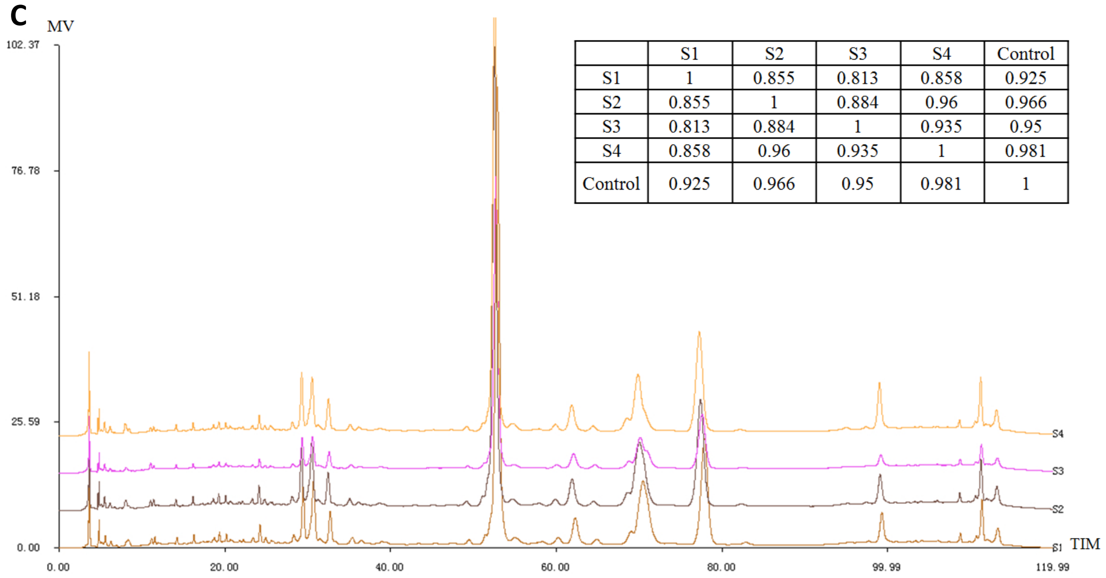

	S1	S2	S3	S4	Control
S1	1	0.855	0.813	0.858	0.925
S2	0.855	1	0.884	0.96	0.966
S3	0.813	0.884	1	0.935	0.95
S4	0.858	0.96	0.935	1	0.981
Control	0.925	0.966	0.95	0.981	1

Fig.S4. Quality control of Ban Xia Xie Xin decoction. A：HPLC graphs of main chemicals in extract preparations at 280nm; B : HPLC graphs of main chemicals in extract preparations at 210nm; C: HPLC graphs of Ban Xia Xie Xin water extract preparations after 7 days of storage and between 2 batches. (S1: the first batch herbs water extract; S2:S1 stored for 7 days at 4℃ ; S3: the second batch herbs water extract; S4: S3 stored for 7 days at 4℃.) The table shows the similarity of each group.

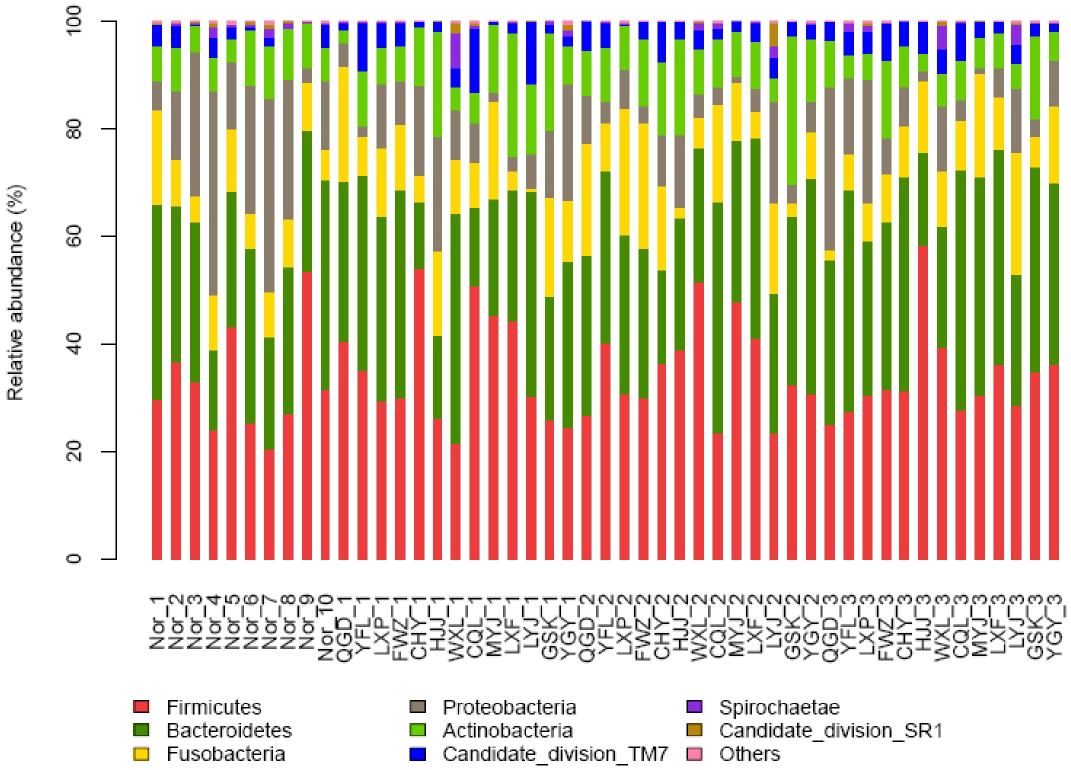

Fig.S5. 16S rRNA sequencing analysis and taxonomy classification of the tongue coating microbiome at the phylum level(All 4 groups)

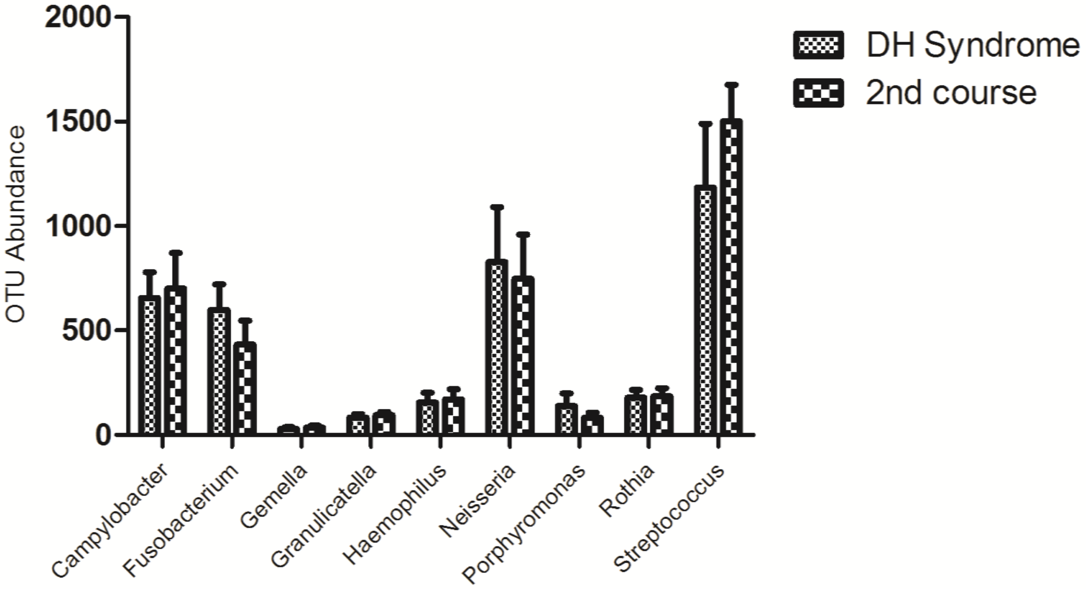

Fig.S6. Abundance of OTUs in patients with CEG yellow tongue coating and 2nd course treatment group (No significance were found)(“YTC”:yellow tongue coating)

Supporting Tables

Table S1. Demographic information of the study participants in discover phase

Demographic variable	Healthy volunteers **(n=10)**	CEG patients with yellow tongue coating **(n=13)**
Age(Mean ±SD)	27.7 ±7.9	52.4 ±11.6
Male(%)	3(30)	7(53.8)
Female(%)	7(70)	6(46.2)
Smoking(%)	0	2
Helicobacter pylori（%）	0	0

Table S2 Demographic information of the study participants in validation phase

Demographic variable	Healthy volunteers **(n=11)**	Patients from Gastroenterology dept **(n=26)**		Patients from Cardiovascular dept **(n=14)**	
	White tongue coating only	Yellow tongue coating (n=16)	White tongue coating (n=10)	Yellow tongue coating (n=6)	White tongue coating (n=8)
Age(Mean ±SD)	54.9 ±5.1	58.6 ±10.3	56.6 ±9.1	65.8 ±11.7	68.6 ±13.9
Male(%)	1（9.09）	5（31.25）	6（60）	3（50）	2（25）
Female(%)	10（90.91）	11（68.75）	4（40）	3（50）	6(75)
Smoking(%)	0	3(18.75)	0	0	2(25)

Table S3 The results of staining microscopy

NO.	Disease	Tongue coating figure	Tongue coating color	Phe agar	Gram-staining	Bacillus positive/negative
GI-1	chronic atrophic gastritis	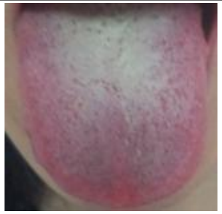	yellow	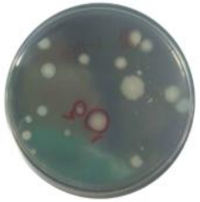	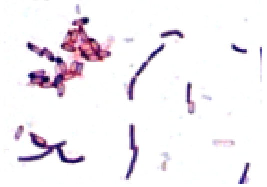	positive
GI-2	gastritis, bile reflux	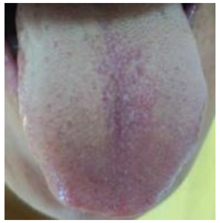	yellow	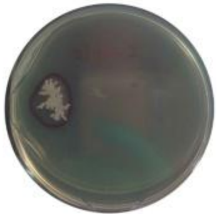	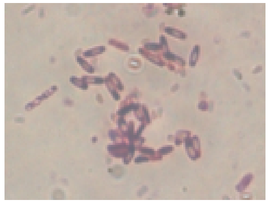	positive
GI-3	chronic gastritis	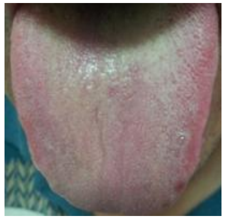	yellow	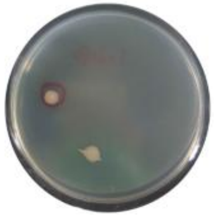	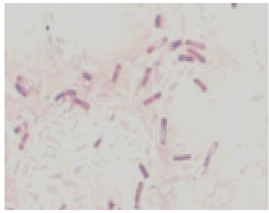	positive
GI-4	chronic erosive gastritis	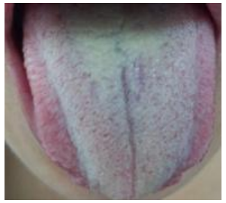	yellow	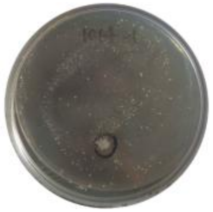	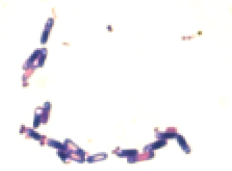	positive

GI-5	chronic atrophic gastritis	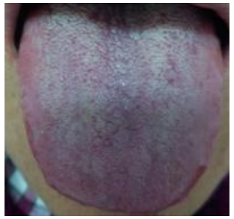	yellow	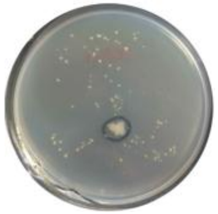	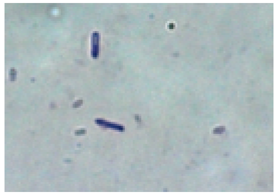	positive
GI-6	chronic atrophic gastritis	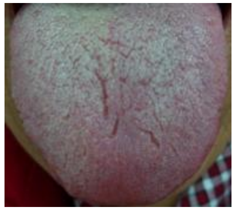	yellow	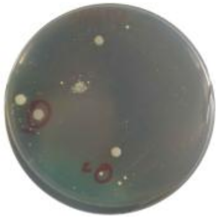	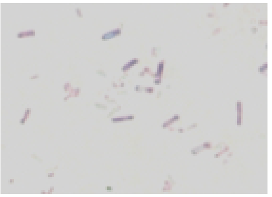	positive
GI-7	chronic atrophic gastritis	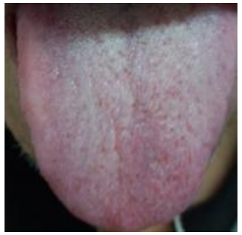	yellow	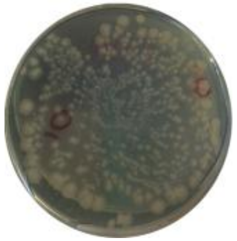	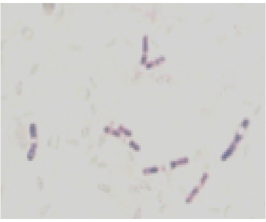	positive
GI-8	chronic atrophic gastritis	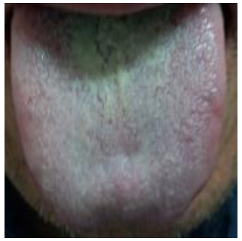	yellow	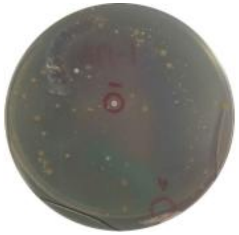	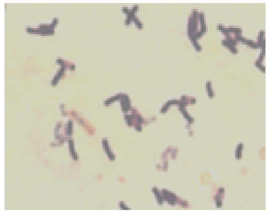	positive

GI-9	chronic erosive gastritis	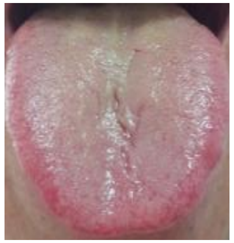	yellow	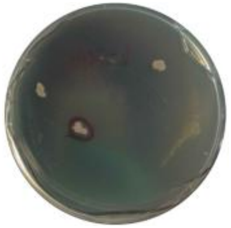	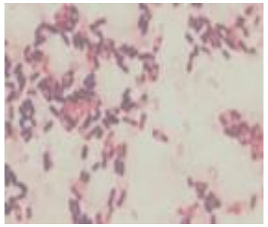	positive
GI-10	chronic atrophic gastritis	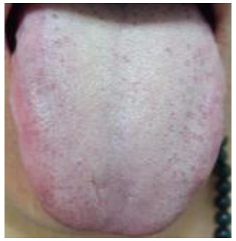	yellow	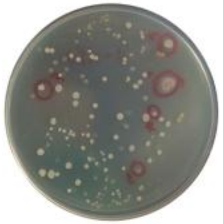	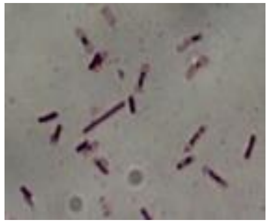	positive
GI-11	superficial gastritis	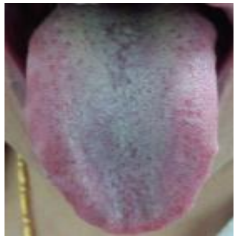	yellow	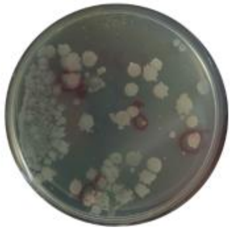	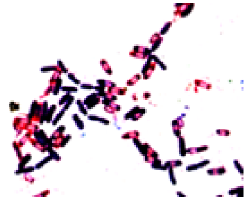	positive
GI-12	gastric polyposis,duodenal bulbar inflammation	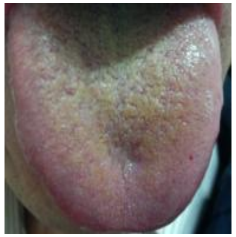	yellow	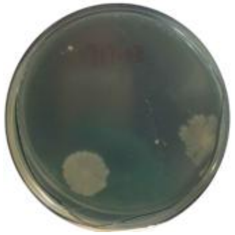	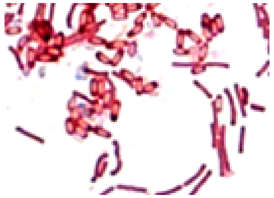	positive

GI-13	chronic atrophic gastritis	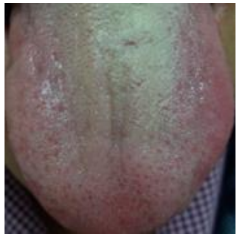	yellow	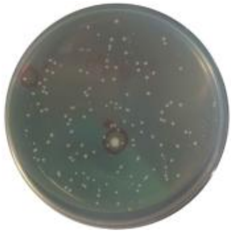	\	negative
GI-14	chronic erosive gastritis	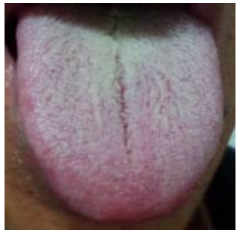	yellow	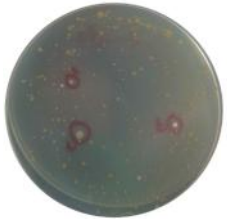	\	negative
GI-15	chronic atrophic gastritis	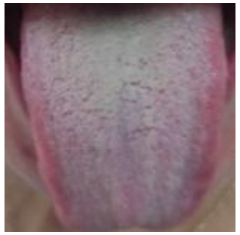	yellow	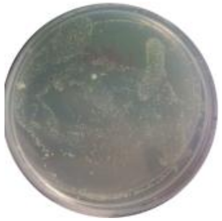	\	negative
GI-16	chronic erosive gastritis	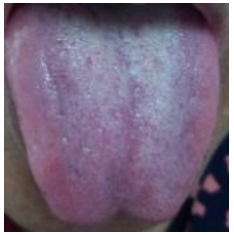	yellow	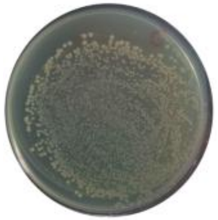	\	negative

GI-17	superficial gastritis,gallbladder polyps	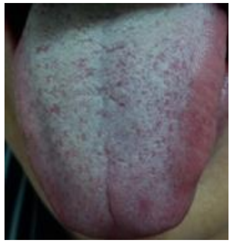	white	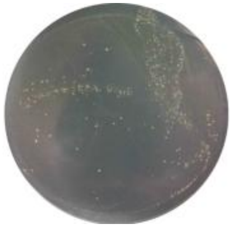	\	negative
GI-18	bile reflux.Ileocecal resection	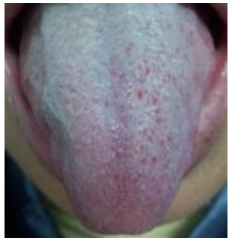	white	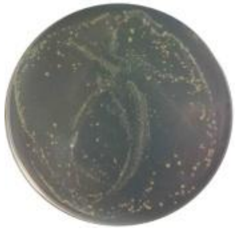	\	negative
GI-19	superficial gastritis	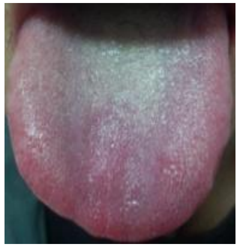	white	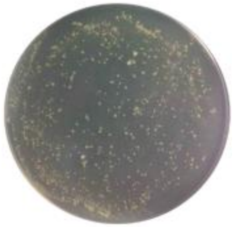	\	negative
GI-20	gastric ulcer	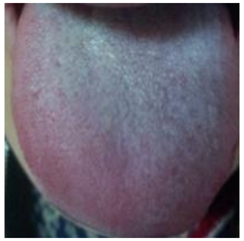	white	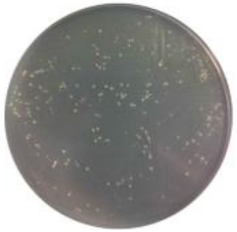	\	negative

GI-21	chronic atrophic gastritis	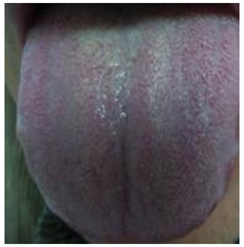	white	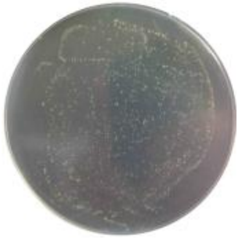	\	negative
GI-22	enteritis	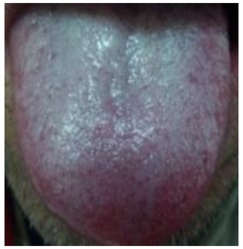	white	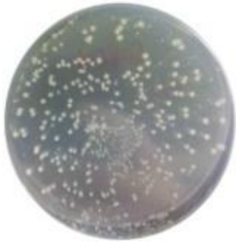	\	negative
GI-23	chronic atrophic gastritis	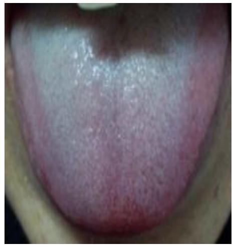	white	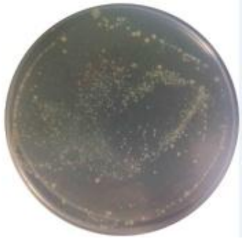	\	negative
GI-24	chronic atrophic gastritis	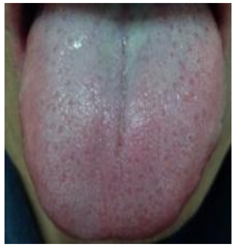	white	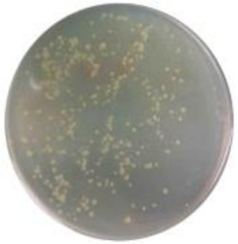	\	negative

GI-25	chronic atrophic gastritis	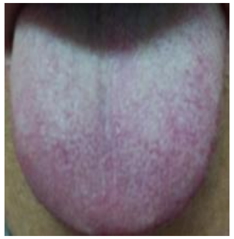	white	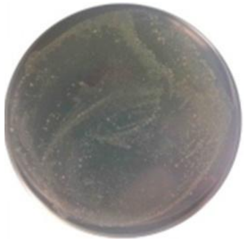	\	negative
GI-26	gastric ulcer	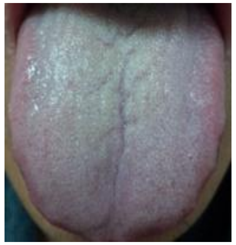	white	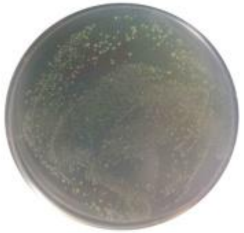	\	negative
Ca-1	Coronary heart disease	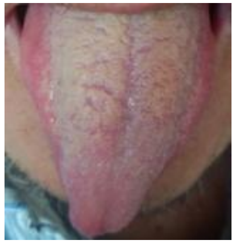	yellow	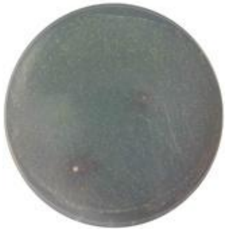	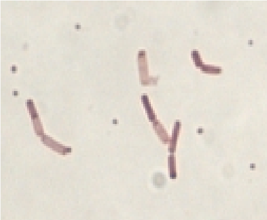	positive
Ca-2	Coronary heart disease	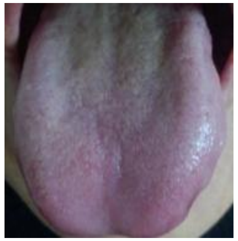	yellow	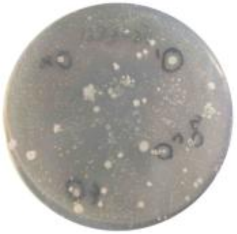	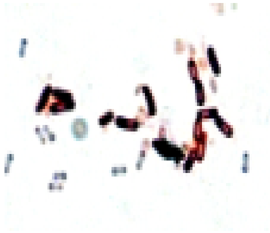	positive

Ca-3	chronic stroke	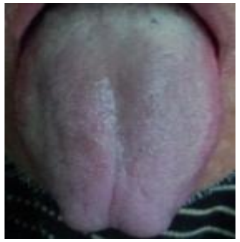	yellow	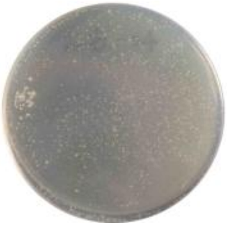	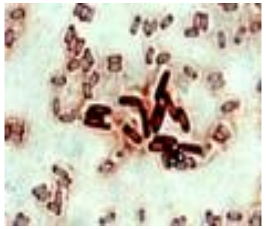	positive
Ca-4	Coronary heart disease	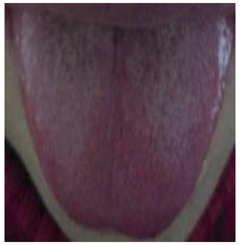	yellow	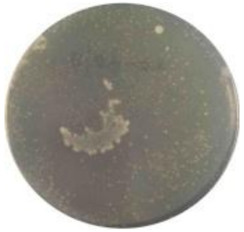	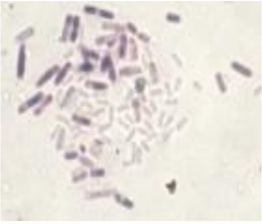	positive
Ca-5	Coronary heart disease	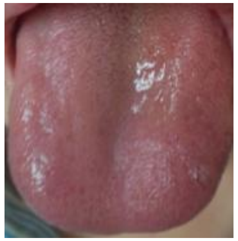	yellow	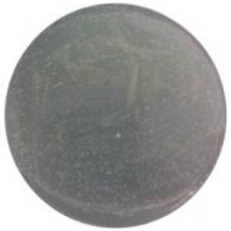	\	negative
Ca-6	Coronary heart disease	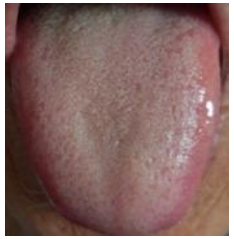	yellow	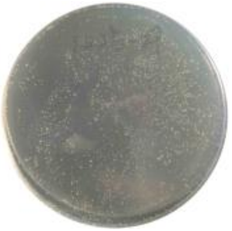	\	negative

Ca-7	Coronary heart disease	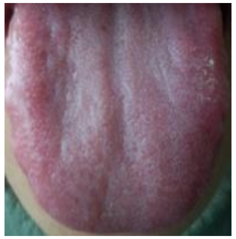	white	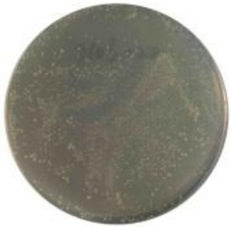	\	negative
Ca-8	miocardial infarction	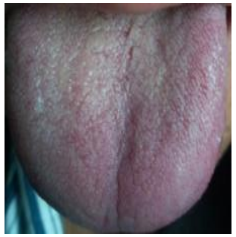	white	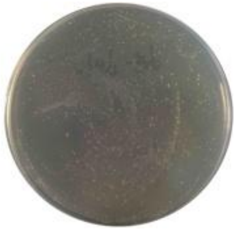	\	negative
Ca-9	Coronary heart disease	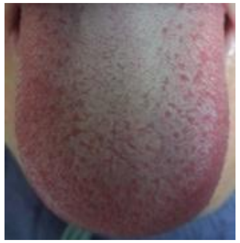	white	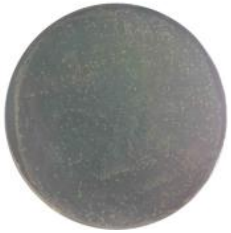	\	negative
Ca-10	hyperuricemia	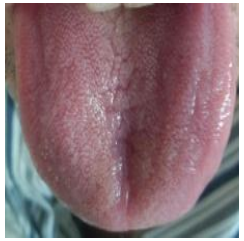	white	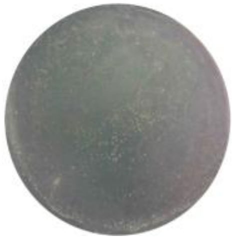	\	negative

Ca-11	arrhythmia	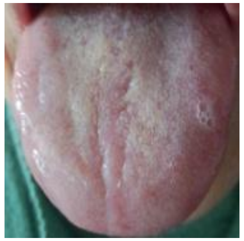	white	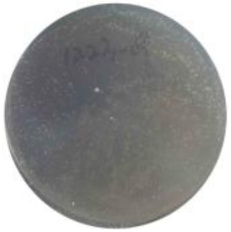	\	negative
Ca-12	Coronary heart disease	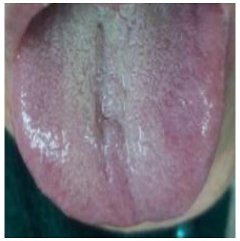	white	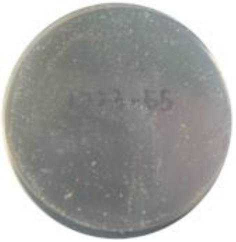	\	negative
Ca-13	Coronary heart disease	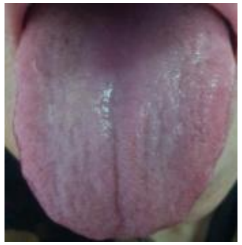	white	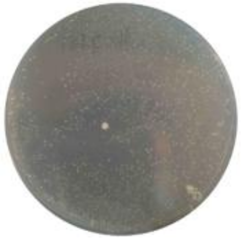	\	negative
Ca-14	Coronary heart disease	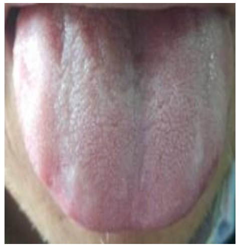	white	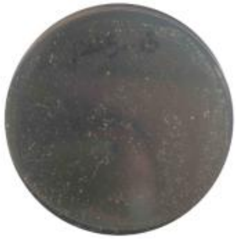	\	negative

H-1	\	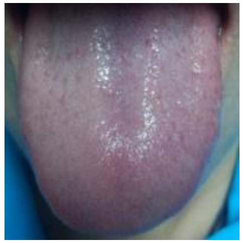	white	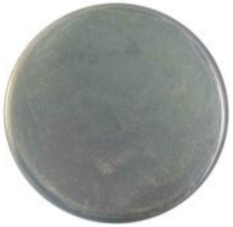	\	negative
H-2	\	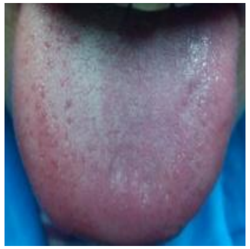	white	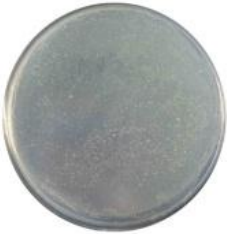	\	negative
H-3	\	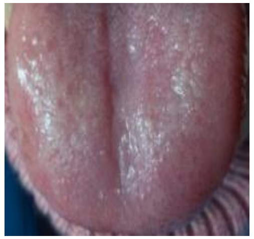	white	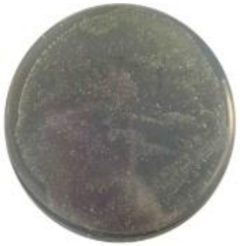	\	negative
H-4	\	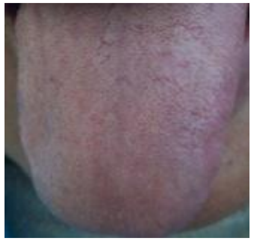	white	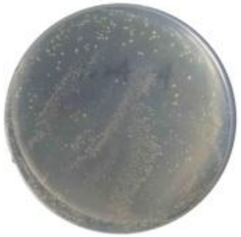	\	negative

H-5	\	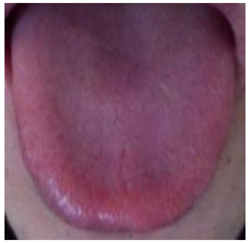	white	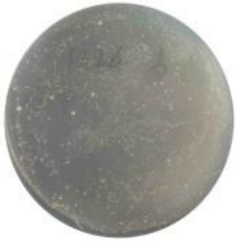	\	negative
H-6	\	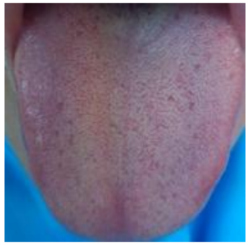	white	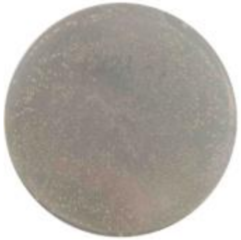	\	negative
H-7	\	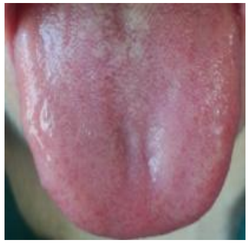	white	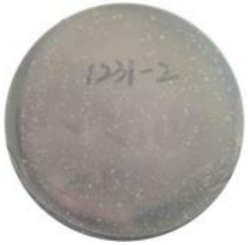	\	negative
H-8	\	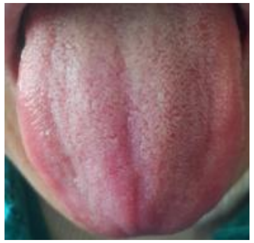	white	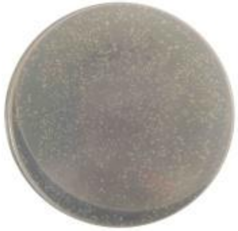	\	negative

H-9	\	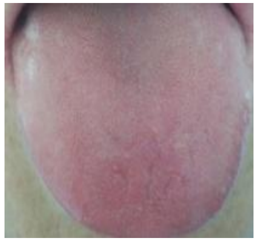	white	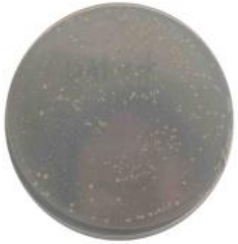	\	negative
H-10	\	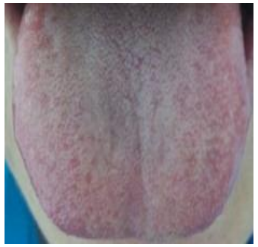	white	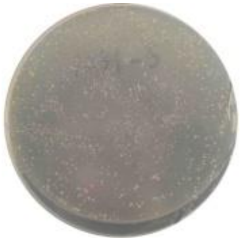	\	negative
H-11	\	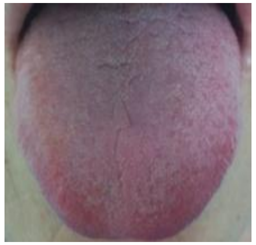	white	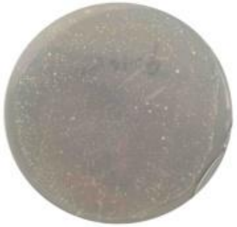	\	negative

Note:"GI-":Samples from Gastroenterology department; "Ca-":Samples from Cardiovascular department; "H-":Samples from healthy volunteers
